# Research Advances in the Use of Bioactive Compounds from *Vitis vinifera* By-Products in Oral Care

**DOI:** 10.3390/antiox9060502

**Published:** 2020-06-08

**Authors:** Cătălina Bogdan, Anca Pop, Sonia M. Iurian, Daniela Benedec, Mirela L. Moldovan

**Affiliations:** 1 Department of Dermopharmacy and Cosmetics, Faculty of Pharmacy, “Iuliu Hațieganu” University of Medicine and Pharmacy, 12 I. Creangă Street, 400010 Cluj-Napoca, Romania; catalina.bogdan@umfcluj.ro (C.B.); mmoldovan@umfcluj.ro (M.L.M.); 2 Department of Toxicology, Faculty of Pharmacy, “Iuliu Hațieganu” University of Medicine and Pharmacy, 6 L. Pasteur Street, 400349 Cluj-Napoca, Romania; 3 Department of Pharmaceutical Technology and Biopharmacy, Faculty of Pharmacy, “Iuliu Hațieganu” University of Medicine and Pharmacy, 41 V. Babeș Street, 400012 Cluj-Napoca, Romania; sonia.iurian@umfcluj.ro; 4 Department of Pharmacognosy, Faculty of Pharmacy, “Iuliu Hațieganu” University of Medicine and Pharmacy, 12 I. Creangă Street, 400010 Cluj-Napoca, Romania; dbenedec@umfcluj.ro

**Keywords:** *Vitis vinifera*, by-products, oral health, antimicrobial, anti-inflammatory, antioxidant, mouthwash, toothpaste

## Abstract

Oral health is considered an important factor of general health and it contributes to the quality of life. Despite the raising awareness of preventive measures, the prevalence of oral health conditions continues to increase. In this context, a growing interest in investigating natural resources like *Vitis vinifera (V. vinifera)* phenolic compounds (PhCs) as oral health promoters has emerged. This paper aims to review the evidence about the bioactivities of *V. vinifera* by-products in oral health. Up to date, a high number of studies have thoroughly reported the antimicrobial and antiplaque activity of *V. vinifera* extracts against *S. mutans* or in multi-species biofilms. Moreover, the bioactive compounds from *V. vinifera* by-products have been shown to modulate the periodontal inflammatory response and the underlying oxidative stress imbalance induced by the pathogenic bacteria. Considering these beneficial effects, the utility of *V. vinifera* by-products in the maintaining of oral health and the necessary steps towards the development of oral care products were emphasized. In conclusion, the high potential of *V. vinifera* by-products could be valorized in the development of oral hygiene products with multi-target actions in the prevention and progression of several oral conditions.

## 1. Introduction

According to the World Health Organization, oral health is an important element of general health and is crucial for the quality of life. Dental caries, periodontal disease, edentulism, and oral cancer are oral diseases/conditions recognized worldwide as a public health issue. Poor mouth hygiene is a key risk factor for oral diseases. Oral diseases and widespread chronic diseases (e.g., diabetes mellitus, cardiovascular diseases, respiratory diseases) share many common risk factors (e.g., sugar rich diet, tobacco use, alcohol consumption) [1]. Moreover, oral infection/inflammation represents a risk factor for the development and/or severity of several systemic diseases [2,3].

In this context, dental hygiene products are deemed essential for oral health. Historically, the first dental cream, containing the powder of oxen hooves, ashes, eggshells, and pumice, was invented around 3000–5000 BC by the Egyptians. A few--thousand years later, the Romans and Greeks increased the abrasivity of the toothpaste by adding crushed bones and oyster shells and added the first flavoring agents. During the same period, the Chinese added ginseng and herbal mints to remove bad breath and also to improve palatability [4]. The development of oral hygiene products has continuously changed ever since, and the products became multi-functional due to the addition of various active ingredients and formulation excipients. As consumers become more and more concerned about the quality of life and the potential unwanted side-effects of some chemicals used in products, natural compound-based cosmetics have seen extensive growth. Nowadays, a high number of cosmetic products for oral care are currently available, while the formulations are continuously improved to achieve multiple benefits, better efficacy, and greater bioavailability of active ingredients or sustainable products. 

In the last few years, naturally occurring antioxidants received increased attention not only in academic research, but also in industry field, as these compounds display a wide range of utility in the pharmaceutical and cosmetic industry due to their beneficial effects on general human health. *V. vinifera* by-products represent an easily available and valuable source of natural antioxidants. Grapes are among the largest fruit crops in the world with an annual production of more than 79 million tons in 2018 [5], from which about 80 percent is used in wine production [6]. These activities generate important waste, up to 30% of the total processed mass [7]. While industries focus on reducing the environmental impact of by-product disposal, consumers have become increasingly aware of the need for health products containing natural ingredients. The bioactive compounds found in winery residues represent important candidates for their cytoprotective, anti-inflammatory, and antioxidant effects [8,9]. In this regard, phenolic compounds (PhCs) are considered the most important active compounds, being responsible for most of the health benefits of vine extracts. Many papers reported the antibacterial, antifungal and antiviral activity of PhCs through their direct action against oral pathogens or by the inhibition of virulence factors [10,11]. In addition, *V. vinifera* extracts have been shown to modulate oxidative stress imbalance and the bacterial-induced inflammatory response in periodontal disease (PD) [12]. However, the PhC’s role in preventing oral diseases is far from being completely understood [11]. Many aspects, including the mechanism of action of PhCs in the oral cavity and the complex interaction between PhCs and microbiota need yet to be considered. Moreover, even though the PhC’s effects were thoroughly described both in vitro and in vivo, current evidence on the efficacy of *V. vinifera* extracts in oral care products is scarce. In this context, the purpose of this review is to summarize the bioactivities of *V. vinifera* by-products, emphasizing the role of phenolic compounds for the prevention of oral disease and the treatment of different oral health conditions. Furthermore, key elements of the development of oral hygiene products based on *V. vinifera* by-products, with multi-target actions in PD, were highlighted.

## 2. Active Compounds in *V. vinifera* By-Products

The phenolic compounds include many classes of substances existing in the plant kingdom, which can be divided into simple phenols and polyphenols. The simple phenols include two categories: the coumarins, commonly existing as glycosides, and the phenolic acids, with two subgroups: hydroxybenzoic acids and hydroxycinnamic acids. The polyphenols categories include the flavonoids, the most diverse class, of which the most important are flavones, isoflavones, flavonols, flavanols, flavanones, chalcones and anthocyanins; other phenolic compounds are stilbenes with a structure derived from trans-resveratrol; lignans composed of two phenylpropane units and tannins, which groups the esters of gallic acids, i.e., hydrolyzable tannins and condensed tannins or proanthocyanidins. In regards to *V. vinifera* extracts, phenolic compounds are present in significant amounts in grapes, being the third most abundant constituents [13], mainly distributed in grape skin and seeds [14], but they can also be found in leaves [15] or tendrils [16]. The phenolic content differs according to the variety, degree of maturity and part of the plant studied [17]. The pretreatment of the samples, the extraction technique, as well as the solvent used may have a significant influence on the recovery of PhCs from natural matrices [18]. Even though a high number of studies show that acetone and methanol lead to the highest yield of individual extraction of polyphenols, hydroethanolic mixtures had shown a better recovery rate of total polyphenols [19]. Considering the solvent choice, ethanol is considered the best option for further incorporation of the extracts in oral care products due to its efficiency, low toxicity, and GRAS status. Moreover, ethanol has the advantage of being reusable and environmentally friendly solvent [19,20]. Table 1 presents the main PhCs identified in *V. vinifera* by-products along with the extraction parameters using suitable solvents for cosmetic and pharmaceutical applications.

The flavonoids were identified as the principal phenolic compounds from *V. vinifera* by-products [21]. The main categories of PhCs present in grapes are phenolic acids (most frequently benzoic and hydroxycinnamic acids), simple flavonoids (mainly catechins, anthocyanins, flavonols) as well as tannins and proanthocyanidins [6]. As regards the biological effects, antioxidant activity was attributed to phenolic acids, flavonoids, lignans and stilbenes compounds while the antimicrobial activity is related to both the flavonoid (flavanols and flavonols) and nonflavonoid (phenolic acids and stilbenes) content [22]. 

The main by-products resulting from the winemaking process include grape pomace (10–20% from the entire mass processed) and stems (2–8%). The pomace contains seeds, pulp and skins, stems, and leaves [23] which consists of 30% polysaccharides, 20% pectic acids, 15% proanthocyanidins, proteins and phenols [24]. For this reason, grape pomace was broadly studied, entirely or as individual constituents [7]. The highest concentration of PhCs was reported in stems, skins and seeds [25]. In the vinification process, the PhCs are poorly extracted, over 70% of PhCs remaining in grape pomace [14,25]. The most abundant phenolic compounds in pomace include hydroxybenzoic and hydroxycinnamic acids, anthocyanins, monomeric and oligomeric proanthocyanidins, flavanols, flavonols, and stilbenes [6,7,25,26,27].

The range of seeds varies from 38 to 52% in dry pomace [28]. The content of PhCs is about 7% of the total weight of seeds, of which proanthocyanidins are predominant [8]. Gallic acid and resveratrol are also found in significant amounts [22,29]. In red grape seeds, protocatechuic acid is the most abundant hydroxybenzoic acid, while in white grape seeds, gallic acid and protocatechuic acid were reported in similar amounts [7]. Grape seeds have been reported to have a higher antimicrobial effect than other grape by-products [8]. The antioxidant potential of grape seeds, mainly ascribed to flavonoid content, was extensively investigated by both in vitro and in vivo studies [7]. 

The polyphenolic profile of skins is influenced by both agricultural variables (cultivation practices and cultivar) and ecological variables (soil, climate, geographic origin and exposure to plant pathogens) [13]. Grape skins represent up to 65% of dry pomace [7] and contain mainly phenolic acids, proanthocyanidins, anthocyanins, flavonols, flavanols, and resveratrol [8]. The anthocyanin content from the skin is responsible for the color of red grape cultivars [30]. On the other hand, the antioxidant and anti-glycation effect of grape skins is related to anthocyanin and proanthocyanidin content [7].

Grape stems range between 1.4% and 7.0% of press residue, out of which phenolic content represents about 5.8% [7]. The phytochemical profile of polyphenols from grape stems has shown that the most abundant phenolic compounds are hydroxybenzoic (gallic acid) and hydroxycinnamic acids, flavonols (glycosylated derivatives of quercetin and kaempferol), flavones, stilbenes (*trans*-resveratrol and ε -viniferin) and anthocyanins [31,32,33]. Despite the significant variation of PhC groups determined in grape stems, they display comparable antimicrobial efficacy [31].

*V. vinifera* tendrils have been reported to have considerable amounts of valuable compounds, such as phenolic acids (gallic, protocatechuic, caffeic, ellagic and caftaric acids) and flavonols (rutin), [16,34,35]. Grape leaves are documented for a wide range of compounds of interest: phenolic acids, flavonols, procyanidins, anthocyanins, and tannins [34,36,37]. Flavonol glycosides were detected in higher amounts in leaves than in other by-products [7]. The valuable properties of grape leaves are recognized by the inclusion in pharmacopoeia monographs. Marketed medicinal products containing leaf extracts are also available [36].

Extensive studies on the chemical composition of grape canes have shown that canes could be considered a valuable raw material for isolation of *trans*-resveratrol. Additionally, benzoic and hydroxycinnamic acids, (+)-catechin, (−)-epicatechin and proanthocyanidins are bioactive compounds identified in important amounts [38,39,40,41]. In terms of biological effects, Moreira et al. demonstrated high antioxidant activity of gallic acid, catechin, myricetin and kaempferol-3-*O*-rutinoside from grape canes [42].

## 3. Bioactivity—Oral Cavity Conditions as Targets of Phenolic Compounds

Despite the increasing awareness of the preventive measures, including fluoride exposure, and improved access to oral health services, oral health conditions are still highly prevalent. In recent years, the World Health Organization has endorsed public health interventions aimed at improving oral health, mainly in low-income populations. At the same time, the strategy shifts the approach from invasive dental interventions to prevention or minimum invasive dental treatments. A great number of oral health diseases could be prevented or resolved in their early stages [1]. Preventive strategies aim to control dental plaque, usually through mechanical and antimicrobial approaches. In this context, a growing interest in investigating by-products as an economical source of natural compounds with antimicrobial properties has been noted. The multiple biological effects targeting the prevention and treatment of microbial-mediated oral pathology are further described.

### 3.1. Microbiota and Antimicrobial Activity of V. vinifera By-Products

Oral microbiota is the second most complex microbial environment of the human body after gut microbiota. About 700 types of microorganisms inhabit the oral cavity. Bacteria are the most prevalent, but fungi, viruses, protozoa and archaea are also present in the oral environment. The composition of the microbiota of healthy people from different communities is similar. Dysbiosis, the unbalance of the dynamic equilibrium between commensal microorganism and oral pathogens, may be related to oral or systemic pathologies [11,69]. 

Microbial-mediated oral pathologies are the result of homeostasis disruption due to the interaction between the host resident microbiome, the host susceptibility and the environmental changes [11]. Dental caries and periodontal diseases (i.e., gingivitis and periodontitis) are considered plaque-mediated diseases, probably occurring as the most prevalent infectious diseases affecting humans [70].

Dental plaque (oral biofilm, plaque biofilm) consists of highly organized microbial colonies attached to teeth surfaces [71]. Plaque biofilm is usually localized at the interface of tooth and gingiva and comprises about 50 bacterial species [3,72,73,74]. *Streptococcus mutans* (*S. mutans*), *Streptococcus sobrinus*, *Lactobacillus spp.*, *Streptococcus mitis* and *Streptococcus sanguis* are among the most prevalent bacteria in dental plaque [70]. The microorganisms are embedded in a matrix of excreted polymeric compounds adhering to solid surfaces, in both natural dentition and dental prostheses. Extracellular polymeric substances are mainly composed of exopolysaccharides and proteins, but also of nucleic acids and lipids. The bacteria embedded in the biofilm are able to circumvent the host immune system defense mechanisms [71]. The microbial composition differs between supragingival and subgingival (periodontopathogenic) dental plaque. Supragingival plaque or cariogenic plaque is colonized with facultative anaerobic bacteria, such as *Streptococcus spp* or *Actynomices spp* [75]. 

#### 3.1.1. Dental Caries 

Dental caries is defined as the progressive destruction of hard tissues of teeth (enamel and dentine) by acidic compounds converted from free sugars, together with an insufficient removal of dental plaque and inadequate exposure to fluoride [1,76]. Dental caries of permanent teeth is globally the most prevalent health condition. The destruction process is dynamic, being controlled by the pH value of dental biofilm and characterized by alternating periods of demineralization and remineralization [76]. The development of dental caries involves both acidogenic (acid-producing) and aciduric (acid-tolerating) bacterial species. On these conditions, the variety of microbiota is decreased because of the acidic pH. Dental caries, or tooth decay, is the result of complex poly-microbial activity at the tooth surface, inadequate oral hygiene, and a high frequency of free sugar intake. The decrease of the buffering capacity of saliva together with low pH conditions lead to an increase in the solubility of hydroxyapatite, the main chemical component of tooth enamel and thus, enamel dissolution and tooth decay occur. *S. mutans* and *Streptococcus sobrinus* are the main acidogenic pathogens in the early development of the carious disease. *Streptococcus mutans,* a Gram-positive coccus, is present in over 90% of isolates from human caries [77]. The pathogenicity of *S. mutans* resides in its ability to induce low pH conditions and to modulate sugar metabolism pathways. In the next step, *S. mutans* co-adheres with other pathogens such as *Candida albicans* and spreads in the oral cavity leading to permanent colonization of hard surfaces and gingival areas. The association between *S. mutans* and *Candida albicans* is based on their biochemical characteristics. The significant role of *Candida albicans* in caries development and progression was previously described [78]. *S. mutans* possess a remarkable ability to transport and transform carbohydrates in organic acids and to grow in low pH stress conditions [79]. As the oral environment is changing, the cavitation progresses gradually, the dissolution of hydroxyapatite crystals in enamel and dentin increases and produces deeper cavitation into the tooth [78]. Subsequently, in advanced stages, *Lactobacillus spp*. promotes the progression of enamel destruction through the increased production of lactic acid. During the progression of the disease other bacteria genera are also abundant in caries microbiota: *Bifidobacterium, Selenomonas, Neisseria* or *Scardovia* [11,70]. Recent studies revealed that other bacteria, such as *Prevotella spp.*, *Dialister spp.*, and *Filifactor spp.,* are involved in the occurrence and progression of dental caries [69,80].

#### 3.1.2. Antimicrobial Effects of *V. vinifera* Extracts in Dental Caries

A high number of in vitro studies have thoroughly reported the antimicrobial and antiplaque activity of *V. vinifera* extracts. The antimicrobial activity of wine and *V. vinifera* extracts is mainly due to several phenolic compounds, such as flavanols, gallic acid, hydroxycinnamic acid, *trans*-resveratrol, and epicatechin [81,82]. Concerning the precise mechanism of antimicrobial activity, several hypotheses have been proposed, underlying the role of phenolic acids [83], the inhibition of extracellular enzymes or the complexation of metal ions from bacterial media [84]. For this purpose, several studies were performed to isolate and identify antimicrobial compounds from *V. vinifera* extracts. The study conducted by Rivero-Cruz et al. aimed at the fractionation of hexane- and ethyl acetate-soluble partitions of *V. vinifera* extract and the isolation of antimicrobial compounds, followed by an evaluation of antimicrobial activity on *S. mutans*. Among all the compounds tested, oleanolic acid, oleanolic aldehyde, 5-(hydroxymethyl)-2-furfural (7), and rutin showed an inhibitory effect against *S. mutans* [85].

Of all the *V. vinifera* by-products, grape seed extracts (GSEs) have been extensively investigated. Most in vitro studies investigated the effect of GSEs on bacterial biofilms. As *S. mutans* is considered the key pathogen involved in caries development, most of the studies focus on the activity of *V. vinifera* extracts against *S. mutans* and *S. mutans* virulence factors. Thus, the use of *S. mutans* biofilm models to induce caries-like lesions has been widely used in experimental studies. 

Zhao et al. found that the GSE at 4 mg/mL inhibited the growth of *S. mutans* and biofilm formation, leading to the suppression of acid production and enamel demineralization [86]. Comparable results in terms of antimicrobial activity were obtained for grape pomace extract at a concentration level of 2 mg/mL. Moreover, the grape pomace extract proved to be efficient in the reduction of *S. mutans* biofilms’ adherence in in vitro experimental conditions [87]. Timothe et al. investigated the chemical composition of different varieties of grape extracts and pomace extracts. Despite the high variability of anthocyanins and flavan-3-ol in the samples studied, grape pomace extract proved to be at least as effective as fruit extracts against *S. mutans* virulence factors by reducing the activity of glucosyltransferases, enzymes involved in the synthesis of extracellular polysaccharides. The extracts also reduced bacterial acidogenicity [88].

Even though most studies were performed on a single type of bacteria, namely *S. mutans*, several studies investigated the antiplaque effect of GSE, grape pomace and red wine extract on *Streptococcus sobrinus, Lactobacillus rhamnosus, Actinomyces viscosus, Porphyromonas gingivalis (P. gingivalis)* and *Fusobacterium nucleatum (F. nucleatum)* [87,89,90]. The GSE effectiveness against biofilm formation was maximum at 2 mg/mL, the inhibitory effect increasing in a dose-dependent manner [89]. In a different study, the same authors assessed the antibiofilm activity of GSE alone or in combination with amine fluoride. The combination of 2 mg/mL GSE and 10.2 mg/mL amine fluoride proved to be efficient in reducing the biofilm formation by suppressing bacterial adhesion to the teeth surface [90]. 

The experiments conducted by Munñoz-Gonzalez et al. investigated the antibacterial effect of red wine and different oenological extracts including GSE, on a different biofilm model of supragingival plaque consisting of *Actinomyces oris*, *F. nucleatum*, *Streptococcus oralis*, *S.mutans* and *Veillonella dispar* [91]. Among all of the extracts studied, red wine extract solutions spiked with GSE showed the highest antimicrobial activity. Compared to other wine extracts, the higher antimicrobial activity of GSE can be explained by the high concentration of flavonoids and their derivatives in GSE, which is mainly responsible for the antimicrobial activity [91]. 

The data obtained during in vitro studies are supported by studies on human volunteers that investigated the antimicrobial efficacy of grape-based oral care formulations. The study conducted by Singla et al. assessed the effect of a mouthwash containing 12.5% GSE on oral streptococci count, in children aged 8 to 10 years after using the product twice daily for seven days. The results showing a significant decrease in salivary streptococci count supported the effectiveness of GSE in preventing dental caries [17]. Another in vivo study concluded that oral rinse for 10 min with red wine led to a significant reduction of bacterial adherence to enamel [92]. 

Besides the activity on *S. mutans* of GSE, the potential effect of proanthocyanidins from GSEs on caries-affected dentin was also investigated. The organic matrix of dentine is mainly composed of fibrillar type I collagen (90%) and non-collagenous proteins, such as phosphoproteins proteoglycans that account for about 10% of the matrix. During the cariogenic process, the organic matrix is degraded by proteases secreted by cariogenic bacteria and host-derived enzymes, specifically matrix metalloproteinases (MMPs) from dentine or gingival crevicular fluid [93]. Proanthocyanidins are considered natural cross-linking agents in the collagen from the dentine matrix as they promote the hydrogen bond formation in type I collagen [94]. Firouzmandi et al. analyzed the remineralization of dentin after the treatment with GSE and GSE+ silver diamine fluoride. The results showed that both agents increased the hardness values, but not the elastic modulus of caries-affected dentin [95]. These findings are consistent with the previous studies showing that GSE can improve the mechanical properties of demineralized dentin by stimulating the inter- and intrafibrillar cross-links in the collagen matrix from dentin [96,97,98,99,100,101,102] and by improving resin-dentin bond strength [101,103]. Furthermore, proanthocyanidins from GSE exhibited a significant inhibition, higher than chlorhexidine, on dentine proteases (matrix metalloproteinases and cysteine cathepsins), responsible for progressive degradation of exposed collagen fibrils [94,104]. Thus, through the combined action of proanthocyanidins, the organic matrix of dentine could be preserved, and the remineralization process could be increased [93]. 

These studies support the complex role of *V. vinifera* extracts in processes from the oral cavity and the anticaries effect through different mechanisms, depending on the phytochemical composition of the extracts.

#### 3.1.3. Periodontal Disease

PD represents a chronic medical condition that progresses from gingivitis to the gradual destruction of supporting structures of the teeth, the loss of the alveolar bone and the deepening periodontal pocket, swelling and/or suppuration leading finally to the loss of teeth [12].

Unlike supragingival plaque, periodontal diseases (gingivitis and periodontitis) are directly associated with the proliferation of Gram-negative anaerobic bacteria. The subgingival plaque developed between teeth and gingival crevice is difficult to remove. The gingival crevicular fluid is considered to be the main source of nutrients for the subgingival plaque [105]. It is an inflammatory exudate composed of serum and different tissue breakdown products, inflammatory mediators, serum transudate, subgingival microbial plaque, extracellular proteins [106,107].

The main causes identified are poor oral hygiene, tobacco use and diabetes [1,74]. Recent studies revealed the high prevalence of this disease worldwide, affecting up to 90% of the general population [75]. The milder form of the disease, gingivitis, consists of an inflammation of the gingiva, associated with erythema and bleeding [12]. The proliferation of bacteria in the pathogenic oral biofilm is considered essential for the initiation and progression of the disease [75]. Due to the bacterial factors, pro-inflammatory cytokines are released and the inflammatory cascade is promoted, leading to periodontal tissue destruction [108]. 

Although bacterial proliferation is critical for the initiation of periodontal disease, chronic inflammation plays a decisive role in the progression of the disease through host-mediated destruction of the supporting structures of the teeth. Currently, the PD is rather perceived as the result of an imbalance between bacterial aggression and host response [72].

The etiological agents of periodontitis are *P. gingivalis*, *Prevotella intermedia*, *F. nucleatum*, *Tannerella forsythia*, *Treponema denticola* and *Aggregatibacter actinomycetemcomitans* [72,75]. The progression of PD is the cumulative result of the combined action of these microorganisms, the individual strains alone being less pathogenic. *Porphyromonas gingivalis* is considered the main etiological factor in the pathogenesis and progression of PD. A characteristic bacterial complex recognized as the “red complex” including *Porphyromonas gingivalis, Treponema denticola* and *Tannerella forsythia* has been detected in advanced periodontal lesions. The synergic action of the complex expresses numerous virulence factors, including enzymes, lipopolysaccharides (LPS) and proteins that could trigger periodontium destruction, bone resorption and initiates the host defense mechanism through cytokine production. Another bacterium, *Fusobacterium nucleatum*, is also a key component in the PD due to its capacity to aggregate to *P*. *gingivalis* [109]. In aggressive forms of PD *Aggregatibacter actinomycetemcomitans* was identified as the main specific pathogen [110].

#### 3.1.4. Antimicrobial Effects of *V. vinifera* Extracts in Periodontal Disease

As regards the periodontal pathogens, the antimicrobial activity was investigated using single species or multi-species microbial biofilm. Thus, the antibacterial action of GSE against two of the anaerobic bacteria associated with the periodontal disease, *P. gingivalis* and *F. nucleatum* was reported by Furiga et al. [89] Another study investigated different compounds isolated from Thompson seedless raisins, out of which oleanolic acid, oleanolic aldehyde, and 5-(hydroxymethyl)-2-furfural were shown to suppress the growth of *P. gingivalis* [27]. Recently, the antibacterial activity against *P. gingivalis* and *S. mutans* was reported for tendrils and leaves extracts [34].

The effect of red wine PhCs (caffeic acid and *p*-coumaric acid) and oenological extracts against *P. gingivalis*, *F. nucleatum*, and *S. mutans* was investigated using an in vitro model of human gingival fibroblasts. The results showed the inhibition of bacterial adhesion to human fibroblasts, probably due to a steric impediment for bacterial attachment to cellular receptors. Moreover, the bacterial metabolization of phenolic compounds was found in the case of proanthocyanidins, catechins, and epicatechin and so was a degradation of 3,5-dihydroxybenzoic acid due to both cellular and bacterial activity. The study highlighted the complex action of bacterial combinations as compared to the individual species leading to different transformations depending on the oral environment [111]. The above-mentioned observations were in agreement with the findings of Sánchez et al. which demonstrated the antibacterial activity against *F. nucleatum* in case of GSE, but when tested on total biofilm bacteria, no significant effects were noted [72]. 

Table 2 summarizes the studies investigating the antimicrobial activity of *V. vinifera* extracts against microorganisms involved in dental caries and periodontal disease.

### 3.2. Periodontal Inflammation 

In recent years, the link between oxidative stress and the inflammation observed in PD has gained more attention, as these processes are highly intertwined. The oral cavity is subjected to many pro-oxidative factors resulting from physiological metabolism or through exposure to xenobiotics (microorganism, air pollution, alcohol and tobacco), food, dental treatment or dental materials. Moreover, PD represents a major source of reactive oxygen species (ROS), an increased rate of free radicals and lower antioxidant capacity being detected in plasma, saliva or gingival crevicular fluid of patients with periodontal disease [111,112].

ROS, such as nitric oxide, hydroxyl radicals and superoxide anions, are generated in periodontal tissue by activated phagocytes as a defense mechanism to bacterial aggression. Moreover, the physiologically produced ROS can improve wound healing [113]. NADPH (nicotinamide adenine dinucleotide phosphate) oxidase and purine degradation pathways are responsible for the accelerated ROS production [114] that can exert direct or indirect effects in periodontal tissue. Under oxidative stress, the nuclear factor erythroid 2-related factor-2 (Nrf2) is translocated into the nucleus where it binds to the antioxidant response element of the DNA, regulating the expression of antioxidant enzymes encoding genes [115]. However, in the pathological situation like PD, this defensive mechanism is overwhelmed, and inflammation occurs. If not resolved, chronic inflammation leads to tissue damage that will further activate macrophages, neutrophils, and fibroblasts that further sustain the inflammatory process mediators.

In gingival cells, bacterial components such as LPS and bacterial DNA are detected by CD14 and Toll-like receptor TLR4, key proteins of pathogen recognition that induce the activation of activator protein 1 (AP-1) and nuclear factor-κB (NF-κB) intracellular signaling pathways [108,116]. The central role of NF-κB in many inflammatory diseases is well recognized, as the nuclear translocation of this transcription factor results in the expression of a plethora of pro-inflammatory mediators that underlie the pathophysiological mechanisms present in PD. Even though the activation of this pathway is meant to have a protective effect, the persistence of stimuli can induce a state of chronic inflammation in the gingival tissue. Following the NF-κB translocation, soluble and membrane-bound chemoattractants, such as IL-6, IL-8, ICAM-1 [117], and tissue remodeling enzymes such as MMP, are expressed [118]. Hard tissue remodeling following osteoclast activation and osteogenic differentiation due to Receptor Activator for NF-κB (RANKL) expression has been observed in PD [108,119]. Recruited immune cells, such as mononuclear phagocytes, antigen-presenting cells and specific lymphocytes T, migrate to the inflammation sites and release locally pro-inflammatory cytokines [120]. Other inflammatory mediators, such as chemokines, prostaglandins, and proteolytic enzymes, are released, contributing to the inflammatory cascade. In each stage of the inflammatory process, pro-inflammatory mediators are produced, leading eventually to a chronic inflammatory response, increased osteoclastic activity and the alveolar bone resorption [121].

The pathogens involved in periodontal diseases, tissue destruction with ROS generation and inflammation with clastogenic and metalloproteinase increased activity are all interconnected, forming a vicious circle. 

To prevent periodontitis from spreading, numerous non-surgical treatment strategies are being used. The first step of the treatment consists of a machine-driven or manual instrumentation aimed to reduce the intraoral bacterial load responsible for the increased reactive oxygen species production following the interaction with the polymorphonuclear neutrophils from the gingival tissue. Those interventions must be accompanied by local and systemic antibiotics and/or antimicrobials [122]. However, the bacterial resistance and the medication’s adverse effects tend to limit this approach, highlighting the need for a co-treatment without long-term side effects that could inhibit the bacteria development and decrease the oral oxidative status [123]. Special attention was directed at the extracts from wine industry by-products that demonstrated—so far—various biological activities, including anti-adhesive and antioxidant effects, considered to increase the efficiency of periodontitis treatment.

#### Antioxidant and Anti-Inflammatory Effects of *V. vinifera* Extracts in Periodontal Disease

Bioactive compounds present in natural plant extracts have been shown to modulate the periodontal inflammatory response and the underlying oxidative stress imbalance induced by pathogenic bacteria [124]. The extensive research on green tea PhCs revealed beneficial effects in the management of PD, justifying the need to investigate other natural resources rich in bioactive compounds [125]. Several studies showing the beneficial effects of specific subclasses or individual PhCs in relation to periodontal inflammation have been published. Based on the current knowledge, PhCs proved to be powerful antioxidants in vitro, having the capacity to counter a wide range of free radicals and to inhibit the lipid autoxidation chain reactions. Moreover, they act as antioxidants in vitro by sequestrating Fe^3+^ and thus inhibiting the Fenton reaction. The antioxidant potential is strongly correlated with the structure of the molecule, in terms of hydroxyl groups and their position, making quercetin, 3-hydroxy group flavonol a compound with high antioxidant properties. It should be emphasized that during the ROS neutralization, antioxidant molecules become pro-oxidants when losing an electron, albeit a more stable one. Additionally, antioxidant molecules can become pro-oxidants in reaction with and transition metal ions, thus further nuancing the protective antioxidant effects of PhCs [126,127]. 

Besides the direct action on the neutralization free radicals, measured by the chemical DPPH, ORAC and FRAP assays [128], PhCs restore the redox homeostasis by enhancing the activities of the antioxidant enzymes superoxide dismutase (SOD), catalase (CAT) glutathione peroxidase (GPx) and glutathione reductase (GR) in cellular and animal studies [129,130]. These restorative properties are related to the Nrf-2 transcription factor that modulates the expression of antioxidant molecules and enzymes, subsequent to ROS insults. The Nrf-2 translocation can also be initiated following the interaction with the cytosolic aryl hydrocarbon receptor (AhR), with several flavonols and flavones including quercetin, luteolin and apigenin being confirmed as AhR agonists and inducers of the Nrf-2 pathway [115,131]. Moreover, by favoring the nuclear translocation of the Nrf-2 transcription factor, PhCs inhibit the nuclear translocation of NF-kB, thus inhibiting the pro-inflammatory and the associated pro-oxidative cellular responses [130]. 

Regarding the use of *V. vinifera* extracts in the management of PD, a large body of studies focused on the beneficial effects of resveratrol, as the consumption of moderate quantities of wine was associated with improved cardiovascular status [132]. In this sense, it was shown that resveratrol may reduce the production of pro-inflammatory cytokines in human periodontal ligament cells exposed to *P. gingivalis* [133], reduce alveolar bone loss in an animal model of periodontitis and also decrease IL-1β, IL-6, IL-8 and TNF-α [134]. In a study on human volunteers, undertaken to evaluate the impact of resveratrol supplementation in diabetic patients, it was shown that resveratrol significantly improves the periodontal status [135]. 

For resveratrol extraction, the whole grape is used however, the winery industry is challenged with the disposal of a high quantity of by-products that can be further valued as they represent a rich source of bioactive compounds that could prove their utility in PD management and other inflammation-related diseases [132]. 

GSE represents one of the most studied *V. vinifera* by-products, phytochemical analysis indicating a high content of proanthocyanidins with broad pharmacological activities and therapeutic potential. In addition, other monomeric phenolic compounds such as (+)-catechins, (−)-epicatechin, (−)-epicatechin-3-*O*-gallate were quantified in GSE. Encouraging results for the use of this by-product as an agent in the prevention and/or management of the PD were obtained in non-cellular studies by Furiga et al., who demonstrated a protective activity of the extract against oxidative stress by measuring the scavenging activity of GSE in comparison with vitamin C and E [89,90]. The non-cellular data are confirmed by in vitro studies—the response of murine macrophages stimulated with LPS of periodontopathogens being reduced after GSE exposure. Exposure to GSE inhibited the generation of nitric oxide (NO) and ROS and modulated the expression of nitric oxide synthase [136]. In a comparative study, Anastasiadi et al. evaluated the phytochemical profile of berries, seeds, skins, pomace and stems of four cultivars of *V. vinifera*, and their antioxidant activities in smooth muscle cell culture and reported an improved antioxidant activity for the GSE [137]. Another in vitro study from Dang La et al. reported that macrophage treatment with GSE before the *A. actinomycetemcomitans* LPS stimulation clearly showed a reduction in the activation of NF-κB p65 and AP-1, this suppression being associated with the inhibition of MMP secretion [138]. An in vivo study using rats fed with a high-fat diet, expected to prompt an excess of reactive oxygen species, supplemented with grape seeds confirmed the protective effects against ROS observed in vitro. The authors reported an increase in the antioxidant system with a decrease in the concentrations of plasma and hepatic lipid peroxide [139]. The anti-inflammatory effect of proanthocyanidins from GSE was investigated by Li et al. [140] in an experimental animal model of inflammation. The dose-dependent anti-inflammatory activity observed was explained through different mechanisms: the suppression of inflammatory cytokine production, namely IL-1β and TNF-α, the inhibition of lipid peroxidation and production of Prostaglandin E 2 (PGE_2_) and nitric oxide (NO). Similarly, in the study conducted by Özden [141], the interference of GSE in the inflammatory process from the periodontal tissues was investigated through histomorphometric and immunohistochemical analyses. The histomorphometric parameters showed a lower degree of inflammation as well as the improvement of connective tissue level and bone healing [141]. In line with the reports of the proanthocyanidins from *V. vinifera*, proanthocyanidins from cranberries have been shown to be effective in treating periodontitis by inhibition of: (1) the biofilm formation by *P. gingivalis* and *F. nucleatum*; (2) the adhesion of *P. gingivalis* to different proteins; (3) the growth of *P. gingivalis*, *Treponema denticola* and *Tannerella forsythia* in periodontal pockets; (4) the production of proinflammatory cytokines; (5) the production of MMPs [142].

Stem extract was also investigated for its free radical-scavenging capacity using the DPPH method, the results obtained indicated stems as an inexpensive, abundant and valuable source of natural antioxidant [33,65,143,144]. In addition, grape stem extracts displayed the capability to prevent the oxidation of LDL−lipoprotein at very low concentrations and to decrease the ROS concentration [33]. An increase in the glutathione levels is responsible for the effect of stem extracts on endothelial and muscle cells, a reduction of lipid peroxidation and protein oxidation being observed following the extract treatment, significantly improving the cellular redox status [65].

Important effects that could contribute to the cell protection from oxidative insults were also described after the exposure of normal human keratinocytes to tendril aqueous extract when an increase in the reduced glutathione concentration in a time- and dose-dependent manner was observed [145]. In a comparative study between leaves and tendril extracts, Moldovan at al. reported that the tendril extract exhibited better antioxidant, anti-inflammatory and cytoprotective effects on human gingival fibroblasts [34]. The authors hypothesized that the difference in the observed effects is due to the higher total phenolic content measured in tendrils extract [34]. 

The analysis of a *V. vinifera* root extract indicated a high content of dimeric and oligomeric stilbenoids, partially responsible for the free radical scavenging activity observed. Additionally, an antioxidant activity, characterized by Nrf2 activation with the subsequent initiation of antioxidant genes transcription, was observed after the incubation of Huh-7 cells with the extract. In the same study, the murine macrophages pre-treated with the root extract and further stimulated with LPS, displayed a significant decreased in the NF-κB target genes IL-1β and iNOS on the mRNA level [146].

Grapevine leaves contain more than 200 identified substances, including a high content of flavonoids such as quercetin glucuronide and kaempferol glucosides [36]. Due to their composition, the leaf extract can protect against the oxidation of emulsified linoleic acid and it displays a high free radical scavenging ability, high chelating activity on metal ions and high reducing power [147]. Recent research has shown the effect of grapevine leaves in NF-κB pathway inhibition through the reduction of nuclear factor-κB driven transcription and nuclear translocation in human gastric epithelial cells [148] and in human keratinocytes cell lines [149]. The anti-inflammatory activity of the *V. vinifera* leaves has also been tested in another in vitro study using human keratinocytes exposed to UV radiation. [150]. The possible utility of leaf extract in the management of the PD associated inflammation was recently reported by Moldovan et al. in an in vitro model using human gingival fibroblasts. Exposure to the leaves extract attenuated the pro-inflammatory response after LPS stimulation by decreasing the levels of IL-1β, IL-6 and IL-8. Moreover, the extract displayed a cytoprotective effect, by decreasing the nicotine-induced toxicity, nicotine being one of the most important factors in the development and the progression of PD [34]. The anti-inflammatory effect of grapevine leaf extract was also confirmed by in vivo studies [151].

Grape pomace is another *V. vinifera* by-product with important free radical scavenging activities, able to prevent lipid peroxidation [152]. In this context, a cytoprotective effect of grape pomace in H_2_O_2_ induced oxidative stress was investigated by Maluf et al., demonstrating a beneficial impact even at the lowest concentration tested [153]. These results are in line with the findings of Goutzourelas et al., who demonstrated that, following the exposure to grape pomace extract, the enzymatic activity of gamma-glutamylcysteine synthetase and glutathione S-transferase from endothelial and muscle cells increased [129]. In addition, a regenerative potential of PhCs from grape pomace extract was investigated on human mesenchymal stem cells, the authors describing a decrease of NF-κB ligand activator/osteoprotegerin ratio and an increase of expression of genes involved in osteoblast differentiation [154]. Grape pomace PhCs have been also shown to increase the mechanical properties of pericardium membrane, acting as natural collagen crosslinkers, with relevance to periodontal regeneration [155]. Chidambara et al. support the in vitro findings by revealing a protective effect of the extract in CCl_4_ exposed rats. Co-administration of the extract was associated with a significant improvement of the CAT, SOD, and GPx activities and a decrease of lipid peroxidation to control values [152]. 

Table 3 summarizes both in vitro and in vivo studies investigating antioxidant and anti-inflammatory effects of *V. vinifera* extracts.

### 3.3. Patent Review

The recovery of bioactives from winery waste products is a subject with important economic implications, thus, it is a domain open to technological innovations. Innovations of all kinds are summarized in patents—legal documents that ensure exclusive rights for an invention. They describe products or processes that offer new solutions to particular problems and contain vast amounts of technical information; therefore, patent analysis is a useful tool to summarize the progress made in a specific field [156]. As previously shown, scientific literature provides plenty of data regarding *V. vinifera* parts and the rich composition in polyphenols of winery by-products. Their effects were thoroughly studied both in vitro and in vivo, but there is little scientific information about their inclusion in cosmetic products and especially oral care products. Table 4 describes some examples of patents or patent applications filed for oral care products that include *V. vinifera* extracts.

In 2002, Procter and Gamble Co. filed the patent *Oral Care Compositions* for the development of the so-called portable oral care products that claimed to provide benefits comparable to frequent brushing. The described products are in the forms of dentifrices, mouthwashes and chewing gums. Their effect relies on the synergy between Citrus or Vitis seed or pulp extract with at least 15% polyphenol content, and other oral care actives, as anti-calculus agents, desensitizing agents, anti-plaque agents or oral malodor-controlling agents at ratios between 0.5% and 7%. The particular types of polyphenols responsible for the effects are flavonoids (flavanols, proanthocyanidins, flavanones and flavonols, anthocyanins, anthocyanidins, and anthocyanosides) obtained by solvent extraction using water, alcohols, glycerol, propylene glycol or their mixtures as the solvent. The authors claimed that if products contain less than 10% of water, they preserve their stability over time, without degradation or discoloring phenomena. The described mouthwash contained 10% ethanol, 8% glycerol and about 70% water and the extracts 0.5% were associated with 0.25% zinc chloride. The proposed toothpaste contained 2% extract together with 2% sodium lauryl sulphate, a surfactant with foaming properties, 20% precipitated silica as an abrasive agent and 2% hydrophilic polymers as thickening agents [157].

Vezin applied for a patent in the U.S. in 2008 for the use of an aqueous grape seed extract combined with at least one fluorine salt to combat the formation and accumulation of dental biofilm and compositions comprising said combination. The extracts were obtained from white grape pomace, extracted with sulfurized water, a process that led to a total content of 33.0% oligo-proanthocyanidins and 13.32% epicatechin. The author claimed that the extract proved an anti-adhesion activity, inhibiting the attachment of bacteria cells to the buccal surfaces, without perturbing the equilibrium of the oral ecosystem. The effect of extracts was potentiated by sodium fluoride or fluorinol. The compositions, used as mouthwash, gel, toothpaste, gingival gel or chewing gum, would contain between 1 mg/mL and 3 mg/mL of aqueous grape extract and between 1000 ppm and 1500 ppm of fluorine [159].

LoPesio applied for a patent in 2011, entitled *Multi-purpose dental appliance cleaner*, a composition delivered as a rinse, wet wipe towelette or spray with antimicrobial properties as hygiene products for dental appliances like athletic mouth guards, night guards, orthodontic retainers and teeth whitening trays. The antimicrobial effects are due to plant-based extracts obtained from the seeds, pulp and/or fruit of *Citrus* or *Vitis* which contain quercetin, quercetin glycoside, halperidin, campherol glycoside, apigenin, and dihydrocampherol glycoside, converted to ammonium salts in the extract mixture to improve stability. The plant-based actives are associated with a “generally recognized as safe” cleaning agent. Such cleaning products include acetic acid, glycolic acid, lactic acid and citric acid, as well as hydrogen peroxide or denatured alcohol. The invented product comes as a solution available for use as a rinse, impregnated in towels or used as a spray, containing between 0.5% and 2.5% extract, 0.3–1% glacial acetic acid, 2–20% glycerin, a flavor, a stabilizing agent, a coloring agent and deionized water. The inventor emphasizes the absence of several compounds commonly found in oral care products, thus the reduced risk of adverse events. The bacteria count tests made on dental appliances cleaned with the invented product resulted in up to 99.9% microorganism reduction [158].

A patent on *Oral rinse composition and method* was filed by Shine in 2009 and assigned to Thres Flo, LLC, based on a mixture of active ingredients with claimed effects on various symptoms of periodontal disease. The solution contained one or more essential oils or volatile substances as 0.028–0.03% eucalyptol, 0.012–0.016% menthol, 0.018–0.022% methyl salicylate, 0.019–0.023% thymol or 0.031–0.035% tea tree oil for their antimicrobial and analgesic effects. Hydrogen peroxide as a 3% solution was added as a debriding agent from about 30% to about 35%. Alcohols such as ethanol, 1-propanol, 2-propanol, 1-butanol, 2-butanol, 2-methyl-2-propanol or propylene glycol, were necessary as solvents for the water-insoluble ingredients. Extracts were selected from the group consisting of antioxidants and anti-inflammatory and antimicrobials actives, thus containing phytochemicals such as polyphenols, flavanols, proanthocyanidins. The GSE content was between 0.032% and 0.041% w/v. Nonionic surfactants were recommended for complete dispersion of low water solubility active ingredients, such as poloxamer 407 from 0.03% to 0.18%. The tests performed on patients suffering from various dental conditions revealed important positive effects on bleeding, pain, odor and clean feel [160].

Kamath and Nair applied for a patent in 2006 for the Colgate-Palmolive Company based upon the preparation of a *Red herbal dentifrice*. A blend of botanical ingredients was used at ratios comprised between 1% and 10%, among which was basil oil, black pepper oleoresin, camphor, *Terminalia chebula*, clove oil, ginger oleoresin, neem oil and plant extracts like GSE. The herbal extracts were accounted for their pain-relieving, astringency, antibacterial and anti-inflammatory properties. They were associated with natural calcium carbonate as a mild abrasive, but also with red iron oxide as low abrasive agent. The declared proportion of the components is about 0.5 to 20 parts of herbal blend to 100 parts of calcium carbonate and red iron oxide. The formulations do not exclude other oral care actives like fluoride ion sources, antibacterial agents, enhancing agents, whitening agents, anticalculus agents, antioxidants, sialagogues, breath freshening agents, antiplaque agents, anti-inflammatory agents or desensitizing agents, in the corresponding ratios [161].

In 2003, Mumoli filed a patent for a *Single-dose quick dissolving cleansing agent with medicinal properties*. It contained about 10% to 30% of a surfactant, a mixture of disintegrants up to a total of 15% to 75% and between 3% and 20% phytotherapeutic extract. The product was obtained by compressing the mixture into compacts of 0.3–5 g. The high disintegrant content would promote fast disintegration in contact with water together with a cleansing effect due to surfactants. Upon hydration, the product could be used as toothpaste. The phytotherapeutic actives can be selected from the following list *Betula alba, Aloe ferox, Achillea millefollum, Arnica montana, Calendula officinalis, Fucus vesiculosus, Humulus lupulus, Melissa officinalis, Urtica dioica, Rosmarinus officinalis, Rosa aff. rubiginosa, Salvia officinalis, Sambucus nigra* or *V. vinifera* for their therapeutic or protective effects. *V. vinifera* extract was chosen for the high amount of antioxidants and its vasoconstrictor and astringent tonic effects [163].

An innovative oral care product was described in a patent filed by Markell Hurwitz in 2008, entitled *Oral hygiene tablets and capsules for direct oral delivery of active ingredients*. The tablets or capsules were supposed to be dissolved in the saliva and/or water delivering immediately the actives to the oral cavity. An inner cavity of the tablet/capsule protected by an outer shell would host the active ingredients consisting in mouthwash, toothpaste, mouth soothing and numbing agents or fluoride rinses. The tablet is composed of a mixture of sodium bicarbonate and citric acid to produce effervescence and fast dissolution, flavors, pigments, sweeteners, or binders. Each tablet/capsule would contain between 7 g and 15 g toothpaste or mouthwash, preferably of natural origin and lipophilic character like essential oils and extracts, to avoid the degradation of the outer shell. The grape extract was associated with menthol, and extracts of *Piper cubeba*, *Glycyrrhiza glabra*, *Acorus calamus*, *Alpinia galanga*, *Aloe vera* gel, *Hydrastis canadensis*, *Calendula officinalis* or *Sanguinaria canadensis* [162].

### 3.4. Key-Points of the Oral Care Formulations based on V. vinifera By-Products

#### 3.4.1. Selection of Ingredients for Oral Care

Currently, there is a growing interest to develop formulations with clinically proven efficacy which could prevent and control dental plaque and gingivitis and, at the same time, have favorable organoleptic properties, suitable for mass market applications [164]. The high potential of *V. vinifera* by-products could be valorized in the development of oral hygiene products, as dentifrice and mouthwash with multi-target actions in the prevention and progression of several oral conditions.

In this context, certain features of natural active ingredients need yet to be considered. The efficacy of oral care products mainly depends on the concentration of actives, and their overall composition and stability when all ingredients are combined in the formulation. Despite the promising studies, further research is needed to explain the delivery of PhCs to the oral mucosa level. The interaction between polyphenols and the oral cavity is a complex process, which has not been fully clarified and has bidirectional implications: on one side, PhCs can modulate oral microbiota inducing quantitative and qualitative changes, and on the other side, the microbiota can cause a possible bacterial catabolism [11,165]. For example, in vitro studies on cell cultures showed that flavonol-3-*O*-glycosides can be metabolized by bacteria present in the oral cavity and/or by epithelial cells to corresponding aglycones. Moreover, the polyphenolic compounds are susceptible to degradation in the presence of human saliva in a structure-dependent manner [91,165]. Considering these points, and also the scarce information about in vivo efficacy of *V. vinifera* extracts in oral care, additional experiments should be carried out to elucidate the precise mechanism of PhC interaction with microbiota and the potential implications to be taken into account when developing cosmetic formulations.

As regards in vivo evidence of *V. vinifera* extracts in oral care, Singla et al. have clinically investigated aqueous grape extracts used as actives in alcohol-free mouthwashes. They showed a significant reduction in oral streptococci after 48 h and seven days use at lower ratios when compared to pomegranate and guava extracts [17]. However, clinical studies focus on the activity of concentrated individual extracts or bioactive principles, while cosmetic products are complex formulations with many actives usually used in low concentrations and numerous inactive ingredients [166]. Besides the potential beneficial effects of *V. vinifera* extracts in oral care, in cosmetic development, it must also consider the appropriateness of the chosen excipients for PD. Thus, a prudent approach should consider whether the presence of some widely used ingredients, such as surfactants, abrasives and ethanol in toothpaste is advisable for patients with PD.

Sodium lauryl sulphate (SLS) is a commonly used surfactant in oral care products up to 2.5%. The use of SLS in these preparations may be associated with certain undesirable effects, such as inflammation and desquamation of the oral mucosa [167,168]. A causal link has also been shown between the use of this ingredient in products for oral cavity hygiene and the increased frequency of recurrent aphthous stomatitis in certain patients [169]. It has been observed that SLS may exacerbate oral conditions in case of disruption of the epithelial barrier [170]. Therefore, the use of mild surfactants with a lower irritant potential may be an alternative in the case of periodontal inflammation. Consistent with these observations, a clinical study assessed the effect of two kinds of toothpaste containing Steareth 30, a non-ionic polyethylene glycol ether of stearic acid, and SLS on oral epithelial integrity (desquamation). The results indicated a lower level of desquamation in oral mucosa following the use of the toothpaste containing Steareth 30 [170]. Other surfactants that are commonly mentioned in toothpaste composition are cocamidopropyl betaine, sodium methyl cocoyl taurate and less commonly, sodium C14–16 olefin sulfonate and sodium C14–17 secondary alkyl sulfonate. More recently, coco glucoside and decyl glucoside were mentioned in natural dentifrices as alternatives to SLS [4,171,172].

Abrasive agents are ingredients typically present in toothpastes that can increase the abrasive action of tooth brushing [173]. The abrasivity of dentifrices for adult use, quantified through the Relative dentin abrasivity scale, must have a value below 250 to be regarded as safe and efficient [168]. If not mechanically removed using “calculus control” or “tartar control” toothpaste, dental plaque could calcify. The abrasivity of toothpastes should be appropriate to remove dental plaque, but the effect of the abrasives on tooth structures should be considered because of the risk of dental abrasion or gingival lesions [174]. Concern is growing around patients with exposed root surfaces. Moreover, the abrasive tooth brushing is incriminated in the gingival recession and the loss of attached gingiva [173]. Each abrasive has a hardness value influenced by the particle size and morphology. Abrasives used in dentifrices must have hardness values up to three on the Mohs scale, about half of the enamel’s hardness and equal or slightly bigger than dentine’s. Abrasives with smooth surface particles smaller than 20 µm are usually preferred in toothpastes, avoiding rod and needle-shaped particles. Silica, silica hydrate and calcium carbonate are the most commonly used abrasives, at concentrations between 8 and 20%, sodium bicarbonate may be added in concentrations up to 50%; calcium phosphate dibasic and calcium phosphate dibasic dehydrate are also used usually in combination with other abrasives, at concentrations up to 50% [4,175].

The prevention and therapy of PD aim to remove or decrease the plaque biofilm around the periodontium. In this regard, the use of mouthwashes containing chemical inhibitors of plaque biofilm is necessary to control the microbial biofilm [173]. In mouthwashes, the ethanol is usually used in concentrations of 6–26.9% as the solvent, as well as antiseptic and preservative [167]. The presence of ethanol in the mouthwashes is associated with poor palatability of the cosmetic product, a possible irritating effect, mucosal pain and dryness and degradation of composite dental materials [176,177]. Alcohol-free mouthwashes are recommended in patients with xerostomia [178], smokers [176] or mucosal injuries [179]. In vitro studies have shown that ethanol can increase the penetration of carcinogenic substances through the oral mucosa present in tobacco smoke [177]. Although the studies that have been undertaken so far failed to find a statistically significant association between mouthwash use and risk of oral cancer [178,180,181], mouthwash use may act as an effect modifier of tobacco smoking [180]. This is a major concern since tobacco smoking is considered the external factor with the greatest impact on the development and the progression of PD, being associated with high loads of periodontal pathogens, but also to impaired immune host responses and slow clinical healing processes [182]. A dose-dependent relation was confirmed between the number of cigarettes smoked per day and the probability of periodontitis occurrence [182]. Out of the thousands of substances identified in tobacco smoke, nicotine is the most pharmacologically active, reported for free radical production with consequences on gingival and periodontal ligament fibroblast functions [183]. The cytoprotective effect of PhCs was confirmed by in vitro studies on cells exposed to nicotine [34,182,184]. Given the possible implications of tobacco smoking with the concomitant use of mouthwashes containing ethanol in PD, the use of alcohol-free formulations containing PhCs may be considered due to the protective effect on gums.

#### 3.4.2. Formulation Design of Oral Care Products

Although the in vitro results support the bioactivity of natural compounds applied in periodontal disease, their effect when incorporated into cosmetic products depends on many variables. The formulation of toothpastes includes numerous inactive ingredients, with different functions, as thickening agents (xanthan gum, carrageenan), humectants (glycerin), abrasive bases (calcium carbonate, silica, hydrated silica), foaming agents (lauryl glycoside, sodium lauroyl sarcosinate, and sodium lauryl sulphate), preservatives (sodium benzoate), sweeteners, flavors and water as the solvent. The mouthwashes also contain a plethora of inactive ingredients like solvents (water, ethanol, glycerin, propylene glycol, PEG 40), surfactants (PEG-40 hydrogenated castor oil), preservatives, sweeteners and flavors [166,185]. All these compounds in both kinds of toothpastes and mouthwashes can influence the release of actives and impact the quality of the product. A rational strategy is necessary when choosing the actives and their doses, as well as the associations of excipients, with a focus on the Quality Target Profile of the desired product, its addressability and expected effects.

The development of oral care products as part of the general category of cosmetics is usually performed in an empirical way, guided by formulation principles such as those previously described, clinical evidence and the formulator’s expertise. However, Regulation EC N° 1223/2009 emphasizes the need for safe cosmetic products, constant quality assurance and compliance to Good Manufacturing Practices. Moreover, the Product Information File (PIF), mandatory documentation when a cosmetic product is placed on the market contains safety and quality issues whose assurance demands previous planning in the formulation and development phases [186]. Quality by Design (QbD) is a concept applied in several fields of industry, including the pharmaceutical industry, which aims to identify, analyze, and manage all sources that might impact the quality and safety of a product. In this context, the application of the QbD concept to oral care product development leads to an easy and cost-effective development and manufacturing process with consistent and quality products and robust processes. Thus, the development of oral care products should consider all the variables that contribute to the quality of the final product. The Design of Experiments (DoE), as a tool of QbD strategy, can be used to develop cosmetic formulations. DoE represents a systematic study based on statistical models that enables the best ranges for the formulation factors with a minimal number of experimental runs [187]. The optimal formulation resides in a Design Space, an area in the experimental domain where all the conditions requested by the Quality Target Product Profile are fulfilled [188,189]. A coherent control strategy, included in the key points of the process, grants the flexibility of the manufacturing and the constant quality of the cosmetic product. So far, the DoE methodology has proven its efficacy in cosmetic formulation, and also in finding the optimal conditions for PhC extraction from winery by-products [41,45,47,51,67]. Thereby, the formulation obtained attains physicochemical and organoleptic characteristics that could contribute to the in vivo performance of the product.

## 4. Conclusions and Perspectives

The novelty of this work not only consists of finding new exploitation directions of *V. vinifera* by-products based on recent progress made in this field, but also in defining the scientific framework for the development of oral care formulations based on *V. vinifera* by-products.

Despite the high number of studies showing that winery by-products are a widely available source of bioactive phytocompounds, especially natural antioxidants, these products are currently underexploited in the cosmetic field. Only a few applications in oral care have been found so far, and additional research is needed to achieve full benefits. In our opinion, for the efficient exploitation of *V. vinifera* by-products, future research should focus on several points:

- The use of emerging techniques with a low environmental impact and sustainable production costs for extract preparation.
- The use of GRAS solvents to obtain low toxicity and biocompatible extracts suitable for oral care products.
- The use of a systematic approach in cosmetic manufacturing that allows for the time-effective and cost-effective development of oral care products.
- Further studies should be conducted to assess the complex interaction between PhCs and inactive ingredients, and also between polyphenols and oral mucosa.
- The development of formulations that fulfill organoleptic characteristics required by consumers.
- The development of highly effective and cost-effective formulations that justify the recycling process.

The rising awareness concerning the environmental impact of by-products has led to new research, aimed towards strategies to reduce waste disposal. In this sense, the sustainable reuse of grape by-products in pharmaceutical or cosmetic sectors could represent alternative approaches. *V. vinifera* is a rich source of bioactive compounds since over 70% of PhCs remain in grape pomace during the winemaking process. The current work reviewed the evidence about the bioactivities of *V. vinifera* by-products in oral health, emphasizing the applicability in oral hygiene products. Thus, several studies supporting the antimicrobial and antiplaque activity of *V. vinifera* extracts against *S. mutans* or in multi-species biofilms were presented. Moreover, the role of the bioactive compounds from *V. vinifera* by-products, concerning the periodontal inflammatory response and the underlying oxidative stress imbalance, was investigated. Considering the complex biological effects of PhCs in the prevention and treatment of microbial-mediated oral pathology, PhCs from *V. vinifera* by-products could be used to control periodontal diseases.

## Figures and Tables

**Table 1:** Overview of main bioactive compounds, extraction techniques and solvents of phenolic compounds (PhCs) from *V. vinifera* by-products.

By-Product Type		Extraction Techniques	Solvent	Analytical Methods	Main Compounds	Reference
Pomace	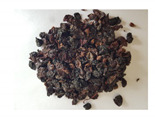	SLE—conventional shaker, UAE, Sequential UAE + SLE	Water 100%, EtOH 100%, Water:EtOH (3:1, 1:1, 1:3)	HPLC-UV/Vis	Phenolic acids: gallic acid, protocatechuic acid, hydroxybenzoic and hydroxycinnamic acids Flavanols: (+)-catechin, (−)-epicatechin Anthocyanins Flavonol derivatives: quercetin, laricitrin and syringetin derivatives Stilbenes: *trans*-resveratrol, piceatannol	[6,7,18,41,43,44,45,46,47,48,49,50,51,52]
		EA, EtOH-based extraction	Ezymatic digestion, EtOH 95% (*v/v*)	HPLC-DAD		
		EA, *DoE*	Aqueous enzymatic digestion, pH, t °C	HPLC-DAD		
		PEF + SLE	Water: EtOH (1:1)	HPLC-UV/Vis		
		SLE	Water: EtOH (1:1), 120 min, 60 °C	HPLC-UV/Vis		
		EA, EtOH-based extraction	Enzymatic digestion or water, EtOH	HPLC-DAD		
		SAS of EtOH extract, *DoE*	EtOH	HPLC-DAD		
		MAE, *DoE*	EtOH 30–60%, 5–15 min	UHPLC-UV/Vis		
		SLE, reflux method *DoE*	EtOH 40–60%, 50–65–80 °C	LC-MS		
		HVED, US-SLE, PEF	Water	HPLC-UV/Vis		
Seeds	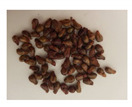	SPE	Water	HPLC-DAD HPLC-MS	Proanthocyanidins Phenolic acids: gallic acid, protocatechuic acid, syringic acid Polyunsaturated fatty acids: linoleic acid, oleic acid, palmitic acid, stearic acid Tocopherols Stilbenes: *trans*-resveratrol	[7,24,26,53,54,55,56,57]
		SLE	70% EtOH, 60 °C, 5 h	HPLC-UV/Vis		
		SLE, maceration 2–10 days	EtOH 0–15% (*v/v*), water	HPLC-DAD		
		SLE	Water, 70% EtOH	HPLC-UV-MS		
Skins	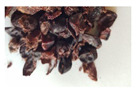	MAE, SLE, *DoE*	EtOH 40–80%, 50–70 °C	UV/Vis	Anthocyanins (red grapes): cyanidin, peonidin, petunidin, delphinidin, and malvidin Procyanidins Phenolic acids: hydroxycinnamic acids, gallic acid Catechins: catechin, epicatechin, epigallocatechin Flavonols: quercetin, quercetin derivatives kaempferol, kaempferol derivatives Stilbenes: *trans*-resveratrol, *trans*-polydatin	[20,25,53,58,59,60,61,62,63,64]
		SLE, UAE, MAE	EtOH 8–92%, solid:liquid ratio (1:3–1:17)	HPLC-DAD		
		PHWE	Water, 40–120 °C, 15 MPa, 3 × 5 min	HPLC-DAD		
		SLE, MAE, UAE	Deep eutectic solvents, SLE-12 h MAE 50–90 °C, 15–90 min UAE 30–90 °C, 15–90 min	HPLC-DAD		
		ASE, SLE, *DoE*	ASE EtOH 20–60%, 5–25 min, 40–80 °C SLE EtOH 49%, 5 h, 50°	HPLC-UV/Vis UHPLC-UV/Vis		
Stems	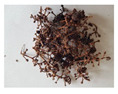	PLE, *DoE*	0–100% EtOH, 40–120 °C, 1–11 min	RP-HPLC-DAD-MS; PLC-UV/Vis	Stilbenes: *trans*-resveratrol, piceatannol Flavanols: (+)-catechin Phenolic acids: gallic acid Procyanidins: procyanidin B3	[19,33,65]
		Superheated liquid EtOH, Supercritical EtOH extraction	EtOH, 60–300 °C, 1 h	Microplate reader (UV/Vis)		
Tendrils	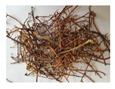	SLE, reflux method	50% EtOH, 60 °C, 30 min	LC-MS/MS	Phenolic acids: gallic acid, protocatechuic acid, caffeic acid, ellagic acid, caftaric acid Flavonols: rutin, quercetin-3-*O*-glucuronide Organic acids: fumaric acid, citric acid	[34,35]
		SLE, reflux method	70% EtOH, 60 °C, 20 h	HPLC-DAD		
Leaves	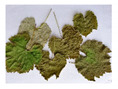	SLE, reflux method	50% EtOH, 60 °C, 30 min	LC-MS/MS	Flavones: quercetin, kaempferol Anthocyans: anthocyanidins, procyanidins Flavonols: quercetol Glycoside flavonoids: hyperoside, isoquercitrin, quercitrin Flavanols: catechin, gallocatechin, epigallocatechin Stilbenes: *trans-*resveratrol	[34,36,37,66,67]
		SLE, kinetic maceration	Water, 25 min	LC/MS-MS UV/Vis-LC-DAD		
		UAE	20–60% EtOH, 30–70 °C, 5–55 min solid:liquid ratio (1:10–1:30)	HPLC, UV/Vis		
		Thermomaceration (skins, seeds, leaves canes), *DoE*	Grape must, 20–60 °C, 0–24 h	HPLC–DAD LC-MS/MS		
Canes	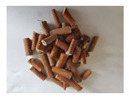	OH, conventional heating	45% EtOH, 80 °C, 20–90 min	UHPLC-UV/Vis	Flavanols: (+)-catechin, (−)-epicatechin Phenolic acids: caffeic acid, trans-*p*-coutaric acid, trans-caftaric acid, gallic acid, syringic acid Stilbenes: *trans*-resveratrol Minerals: K, Ca, Fe, Mg, P, Zn	[39,40,41,68]
		SLE	60% EtOH, 80 °C, 30 min	HPLC-DAD		
		SLE, SLDE, MAE, PLE	Water, SLE 100 °C, 15–60 min SLDE 25−27 °C, 8 bar, MAE 100 °C, 5–15 min, PLE 30–100 °C, 50–100 bar, 10–30 min	HPLC-DAD-MS HS-SBSE-GC-MS		

SLE—Conventional Solid−liquid extraction, UAE—Ultrasound solid−liquid extraction, EA—Enzyme assisted extraction, PEF—Pulse electric field, SAS—Supercritical antisolvent process, MAE—Microwave assisted technology, HVED—High voltage electric discharge, PEF—Pulsed electric field treatment, SPE—Solid phase extraction, PHWE—Pressurized hot water extraction, ASE—Accelerated solvent extraction, PLE—Pressurized Liquid Extraction, OH—Ohmic heating, SLDE—Solid−liquid dynamic extraction, *DoE*—Design of Experiments, EtOH—Ethanol, HPLC—High-performance liquid chromatography, DAD—Diode array detection, MS—Mass spectrometry, UV/Vis—UV/Vis detection, UHPLC—Ultra high-performance liquid chromatography, LC-MS—Liquid Chromatography tandem Mass Spectrometry, RP-HPLC Reversed-phase high-performance liquid chromatography, HS-SBSE Headspace stir bar sorptive extraction.

**Table 2:** Summary of antimicrobial activity of *Vitis vinifera* extracts against microorganisms involved in dental caries and periodontal disease.

By-Product Type	Microbial Strains	Concentration Tested	Reference
Pomace	*Staphylococcus aureus, Bacillus cereus, Escherichia coli, Pseudomonas aeruginosa, Candida albicans, Candida parapsilosis, Candida krusei*	3.9–2000 μg/mL	[82]
	*Streptococcus mutans, Streptococcus sobrinus, Lactobacillus rhamnosus, Actinomyces viscosus, Fusobacterium nucleatum, Porphyromonas gingivalis*	2000–8000 μg/mL	[87]
	*Streptococcus mutans*	62.5–500 µg/mL	[88]
Black grape skin	*Staphylococcus aureus, Enterococcus faecalis, Enterobacter aerogenes, Salmonella typhimurium, Escherichia coli, Penicillium chrysogenum, Penicillium expansum, Aspergillus niger, Aspergillus versicolor*	260 mg, 540 mg, 1080 mg TAE (tannic acid equivalents)/mL	[84]
Thompson seedless raisins	*Streptococcus mutans, Porphyromonas gingivalis*	3.9–500 mg/mL	[85]
Seeds	*Streptococcus mutans, Streptococcus sobrinus, Lactobacillus rhamnosus, Actinomyces viscosus, Porphyromonas gingivalis, Fusobacterium nucleatum*	1000–8000 µg/mL	[89]
	*Streptococcus mutans, Streptococcus sobrinus, Actinomyces viscosus, Lactobacillus rhamnosus, Porphyromonas gingivalis, Fusobacterium nucleatum*	250–8000 µg/mL	[90]
	*Streptococcus mutans*	1–3 mg/mL	[86]
	*Actinomyces oris, Fusobacterium nucleatum, Streptococcus oralis, Streptococcus mutans, Veillonella dispar*	10 g/L	[91]
Red wine extract and seed extract	*Aggregatibacter actinomycetemcomitans, Fusobacterium nucleatum, Porphyromonas gingivalis, Streptococcus oralis, Veillonella parvula, Actinomyces naeslundii*	20 g/L	[72]

**Table 3:** Summary of in vitro and in vivo studies investigating antioxidant and anti-inflammatory effects of *V. vinifera* extracts.

By-Product Type	Type of Assay		Observed Effect	Concentration Tested	Reference
Leaves	in vitro	HGF cell line	↓ ROS production	10–300 µg/mL	[34]
			↓ IL-1 beta, IL-6, IL-8		
		AGS cell line	↓ IL-8, NF-κB Nuclear Translocation	1–100 µg/mL	[148]
		HaCaT cell line	↓ IL-8, NF-κB Nuclear Translocation, VEGF	1–100 µg/mL	[149]
	in vivo	Swiss albino mice	↓ Carrageenan-induced paw oedema	100–400 mg/kg	[151]
			↓ Acetic acid induced vascular permeability		
			↓ Yeast induced pyrexia		
Pomace	in vitro	C2C12 cell line	↑ GCS levels, GST activity	2.5 and 10 µg/mL	[129]
			↓ CAT levels + activity		
		EA.hy926 cell line	↑ GCS levels, GST activity	0.068 and 0.250 µg/mL	
		3T3 cell line	↓ ROS production	0.73–3.65 mg/mL	[153]
		hMSCs	↓ RANKL/OPG ratio	10 and 20 µg/mL	[154]
			↑ BMP2 and Runx2 expression		
	in vivo	Wistar rats treated with CCl4	↑ CAT, SOD, peroxidase activity	50 mg/kg	[152]
			↓ MDA levels		
Root	in vitro	Huh7 cell line	↑ Nrf2 transactivation	1–50 µg/mL	[145]
			↑ HO-1 and GCS		
		RAW264.7 cell line	↓ IL-1*β* and iNOS genes	20 µg/mL	
Tendrils	in vitro	HGF cell line	↓ ROS production	10–300 µg/mL	[34]
			↓ IL-1 beta, IL-6, IL-8		
		NCTC 2544 cell line	↑ GSH levels	12.5–62.5 mg/mL	[145]
Seeds	in vitro	RAW 264.7 cell line	↓ ROS production	0.5–100 µg/mL	[136]
			↓ NO production		
			↓ iNOS expression		
		HVTs-SM1 cell line	↓ ROS production	1–100 µg/mL	[137]
		Monocyte (U937)-Derived Macrophages	↓ MMP-1, -7, -8, -9, and -13 secretion	25–100 µg/mL	[138]
			↓ activation of NF-kB p65		
			↓ AP-1 activation		
			↓ MMP-1 and -9 activity		
	in vivo	Sprague-Dawley rats	↓ lipid peroxide	diet with 5% grape seed	[139]
			↑ hepatic GST activity		
			↑ GSH/GSSG		
		Kunming mice	↓ Croton oil-induced ear oedema	10–40 mg/kg proanthocyanidins fraction	[140]
		Wistar rats	↓ Carrageenan-induced paw oedema		
			↓ MDA, NOS activity, NO, IL-1β, TNF-α, PGE_2_		
		Sprague Dawley rats	↓ Inflammatory cell number	200 mg/kg	[141]
			↑ Connective tissues attachment level		
			↓ Osteoclast density		
Stem	in vitro	HVTs-SM1 cell line	↓ ROS production	1.1–100 µg/mL	[33]
		C2C12 cell line	↓ ROS production	0.95 µg/mL	[65]
			↑ GSH levels		
			↓ lipid and protein peroxidation		
		EA.hy926 cell line	↓ lipid and protein peroxidation	0.20 µg/mL	
			↑ GSH levels		

↓—decrease, ↑—increase, ROS—reactive oxygen species, VEGF—Vascular endothelial growth factor, GCS—gamma-glutamylcysteine synthetase, GST—glutathione S-transferase, RANKL—Receptor Activator for NF-κB, CAT—catalase, OPG—osteoprotegerin, MDA—malondialdehyde, NO—nitric oxide, MMP—matrix metalloproteinase, GSH—glutathione, HO-1— heme oxygenase-1, AP-1—activator protein-1.

**Table 4:** Patents/patent applications for oral care products containing *V. vinifera* extracts.

Type of Oral Care Product	Type of Extract	Bioactive Compounds	Other Actives in the Product	Claimed Effects	Patent/Patent Application Number
Toothpaste Oral rinse	*V. vinifera* seed or pulp extracts	Polyphenols	Potassium nitrate Metal cations salts Polyphosphates, pyrophosphates, phosphonates Fluoride ion source Xylitol	Prevention or treatment of halitosis Antimicrobial effect	US 6,706,256 B2 [157]
Rinse, wet wipe towelettes or spray for dental appliances hygiene	*V. vinifera* seed or pulp extracts	Polyphenols	α- hydroxy- acid, hydrogen peroxide, denatured alcohol, or ethanol	Antimicrobial effect Anti-odor effect Stain remover	US 2012/0207806 A1 [158]
Oral hygiene composition	*V. vinifera* seed aqueous extract	Polyphenols, mainly oligo-proanthocyanidin	Inorganic fluorine salts	Anti-biofilm effect Reduced microbial colonization	US 2010/0129297 A1 [159]
Oral rinse	*V. vinifera* seed extract	Polyphenols	Essential oil Hydrogen peroxide Alcohol	Antimicrobial Anti-inflammatory	US 8,273,385 B1 [160]
Dentifrice	*V. vinifera* extract	Not mentioned	Calcium carbonate Red iron oxide	Astringent effect Antibacterial Anti- inflammatory	US 7,736,629 B2 [161]
Oral hygiene tablets and capsules	*V. vinifera* skin extract	Anthocyanins	Other herbal ingredients	Anti-inflammatory Soothing effect Protective effect on gums and mouth tissue	US 8,728,446 B2 [162]
Quick-dissolving cleansing agent	*V. vinifera* extract	Not mentioned	Sodium fluoride	Antioxidant Vasoconstriction Astringent effect	US 6,664,225 B2 [163]
